# Small cell carcinoma presenting as a biatrial mass with obstructive physiology: a case report

**DOI:** 10.1186/s40959-021-00116-9

**Published:** 2021-08-14

**Authors:** Sara Ratican, Soomin Shin, John Moretto

**Affiliations:** 1 Geisel School of Medicine, Dartmouth College, Hanover, NH USA; 2 California Pacific Medical Center, San Francisco, CA USA

**Keywords:** Cardiac tumor, Small cell carcinoma, EUS-FNA

## Abstract

**Background:**

Small cell carcinoma is a highly aggressive and often fatal cancer that most commonly arises in the lung, although it can occasionally arise from other sites, such as the gastrointestinal tract, prostate or cervix. Cardiac involvement, however, is extremely uncommon and therefore has been poorly documented in the literature.

**Case presentation:**

We describe a rare case of a 31-year-old male with small cell carcinoma presenting as a massive, 15-cm cardiac tumor invading the bilateral atria, interatrial septum, and pericardium without an apparent primary malignancy on PET CT and cardiac MRI. With extensive tissue necrosis, traditional methods of obtaining a right atrial endomyocardial biopsy via internal jugular venous access failed and a diagnosis was made via endoscopic ultrasound guided transesophageal fine needle aspiration of the left atrial mass. Due to the extensive tumor invasion, the patient was not a suitable candidate for surgical resection, debulking, or heart transplant. The patient was treated with etoposide, carboplatin, atezolizumab, and radiation therapy with initial monitoring in the intensive care unit due to concern that tumor lysis may cause rapid cardiac decompensation. Unfortunately, 4 months after chemoradiation therapy, the malignancy progressed and the patient passed away 6 months after the initial diagnosis.

**Conclusion:**

We describe a rare occurrence of small cell carcinoma presenting as a massive cardiac tumor without apparent primary malignancy. This case demonstrates useful alternative diagnostic strategies and treatment considerations for patients presenting with a rare cardiac mass.

## Background

Small cell carcinoma is a highly aggressive and often fatal cancer that most commonly arises in the lung, although it can occasionally arise from other sites, such as the gastrointestinal tract, prostate, or cervix. It most frequently metastasizes to the mediastinal lymph nodes, liver, bone, adrenals and brain [1]. Cardiac involvement is extremely uncommon and therefore has been poorly documented in the literature [1–3]. We describe a rare occurrence of small cell carcinoma presenting as a massive cardiac tumor invading bilateral atria, interatrial septum and pericardium with obstructive physiology and without apparent primary malignancy. This case demonstrates useful alternative diagnostic strategies and treatment considerations for patients presenting with a rare cardiac mass.

## Case presentation

**Fig. 1:**
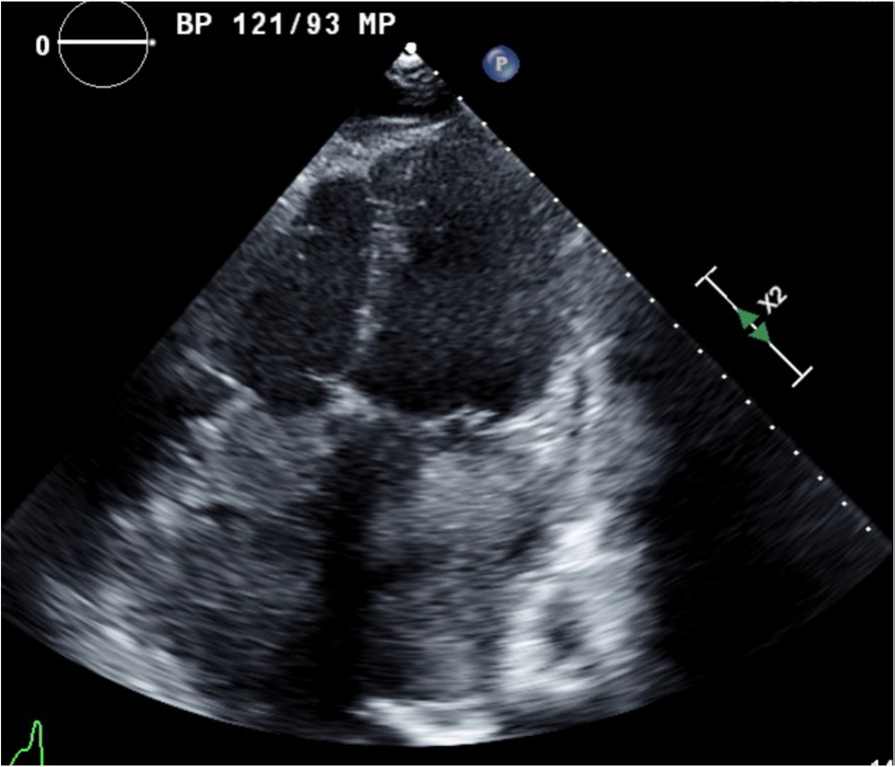
Echocardiogram with apical four-chamber view showing large atrial mass occupying both left atrium and right atrium, obliterating the interatrial septum

**Fig. 2:**
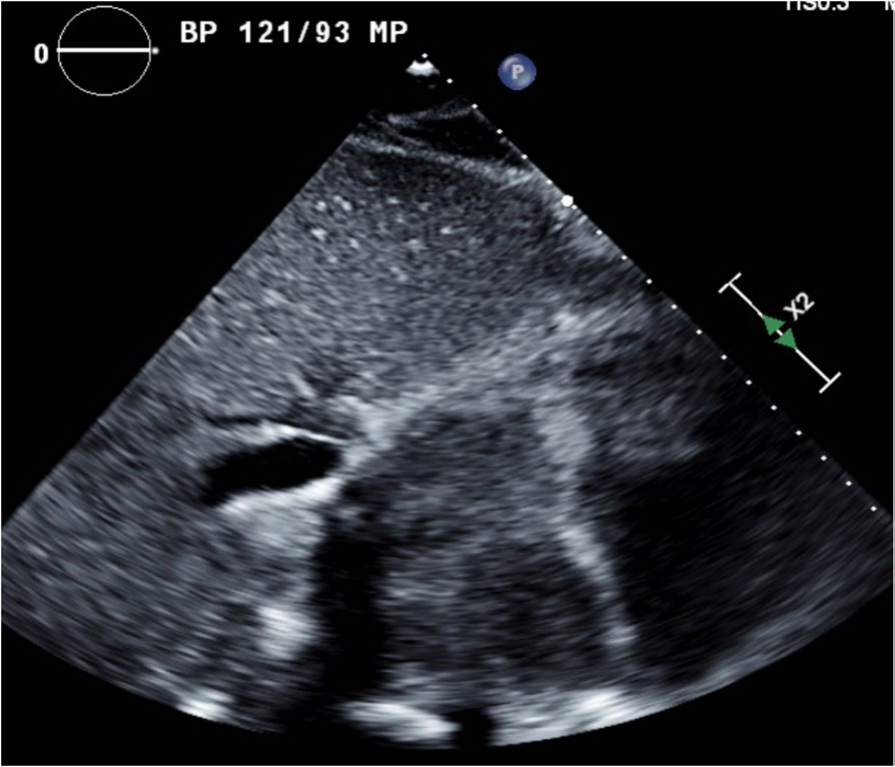
Echocardiogram with subcostal four-chamber view showing large atrial mass occupying both left atrium and right atrium, obliterating the interatrial septum

**Fig. 3:**
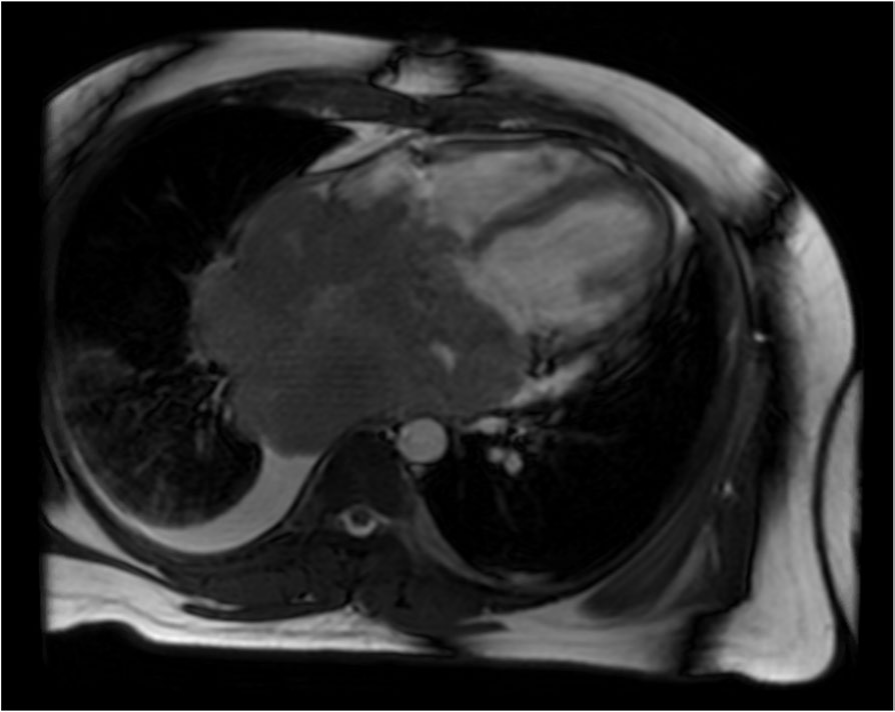
Cardiac MRI with contrast showing neoplasm with biatrial and pericardial invasion, approximately 15 cm in diameter

**Fig. 4:**
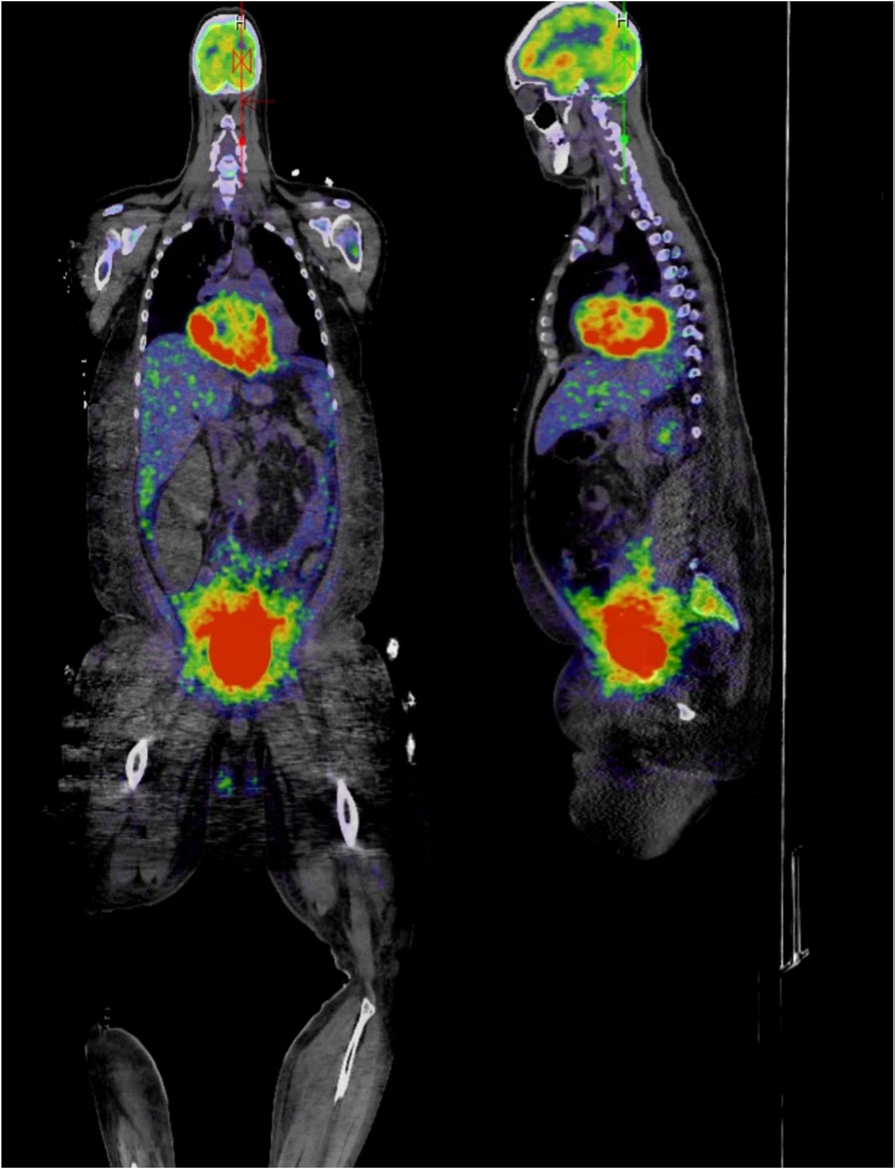
18F-FDG PET/CT scan showing hypermetabolic cardiac mass without alternative primary malignancy

A 31-year-old male with a history significant for exposure to secondhand smoke but no other medical diagnoses presented with shortness of breath for 6 weeks. On exam, he was in atrial flutter with rapid ventricular response and had signs of heart failure with lower extremity edema. CT pulmonary angiogram showed evidence of bilateral pulmonary embolisms and a 15-cm mass occupying both atria, obliterating the interatrial septum, and invading into the pericardium. The features of cardiac mass were further characterized by echocardiogram (Figs. 1 and 2) and cardiac MRI (Fig. 3). PET scan showed hypermetabolic activity of the cardiac mass concerning for malignancy, but did not demonstrate any other areas of hypermetabolic activity (Fig. 4) to suggest an extracardiac primary malignancy.

**Fig. 5:**
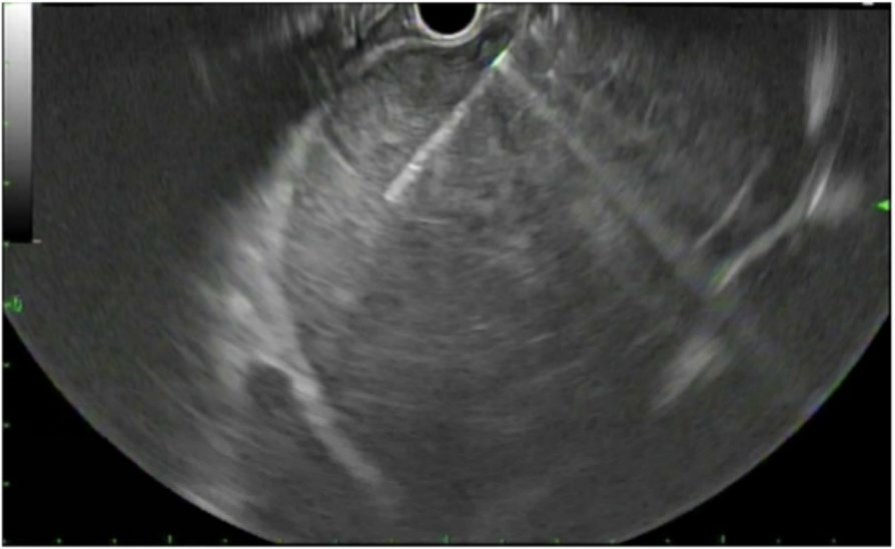
Upper endoscopic ultrasound-guided fine needle aspiration of the cardiac tumor

To further characterize the tumor, we proceeded with numerous attempts for endomyocardial biopsy in the right atrium via internal jugular vein access. Unfortunately, all samples were non-diagnostic and yielded only necrotic cellular debris. Therefore, the decision was made to use an alternative approach, obtaining a left atrial mass biopsy via endoscopic ultrasound (EUS) guided fine needle aspiration (FNA), taking advantage of the fact that the left atrium sits anterior to the esophagus. The patient tolerated the EUS-guided transesophageal endomyocardial biopsy without complications (Fig. 5).

**Fig. 6:**
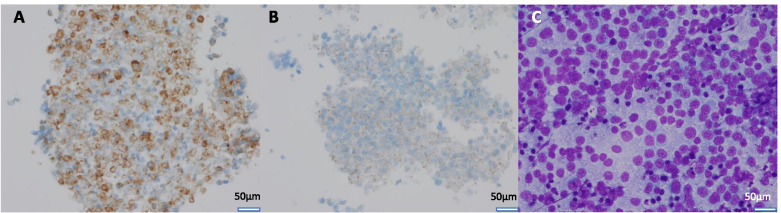
Cardiac Tumor fine needle aspiration (FNA) Pathology. **A** TM Cardiac FNA cell block with synaptophysin IHC stain showing strong positivity, 400x. **B** TM Cardiac FNA cell block with CAM 5.2 IHC dot-like staining, 400x. **C** TM Cardiac FNA Diff-Quik stained smear showing discohesive tumor cells, 400X

The pathology report revealed flow cytometry results, immunohistochemistry findings and cell morphology that were consistent with a diagnosis of small cell neuroendocrine carcinoma. Direct smears and cell block slides showed numerous, loosely cohesive cells with high nuclear-to-cytoplasmic ratio, some nuclear molding and hyper-chromatic nuclei without prominent nucleoli (Fig. 6C). The tumor was positive for synaptophysin, CAM 5.2 and CD56 and negative for CD19 and TTF-1 (Fig. 6A-B). There was no immunohistochemical staining present that would suggest a primary GI tumor origin. While the tumor lacked staining for TTF-1, which is usually present in pulmonary small cell carcinoma, this marker has been found to be negative in up to 17% of cases [4]. Based on the presentation, it was hypothesized that the patient’s cardiac mass was likely a metastasis from a primary pulmonary lesion that was too small to be detected by MRI or PET. However, further evaluation via sputum cytology or bronchoscopy was not performed.

The patient was successfully converted from atrial flutter to normal sinus rhythm on amiodarone. His bilateral pulmonary emboli were effectively treated with an unfractionated heparin infusion, which was later transitioned to dabigatran. Due to the extensive tumor invasion, the patient was not a candidate for surgical resection, debulking, or heart transplant. In the hospital, he was started on etoposide 100 mg/m^2^ for 3 days and carboplatin therapy AUC 5 IV over day 1. The patient’s first round of chemotherapy was monitored in the intensive care unit due to the possibility of tumor lysis leading to rapid cardiac decompensation. The patient tolerated the first round of chemotherapy without complications. Following discharge, atezolizumab (1200 mg IV, 21 day cycle) and radiation therapy (5940 cGy, 33 sessions) were added to his treatment plan. Unfortunately, 4 months after chemoradiation therapy, the patient was found to have extensive epidural involvement with cauda equina syndrome. He was transitioned to hospice and passed away 6 months after the diagnosis.

## Discussion and conclusions

Most cardiac tumors are results of metastatic malignancy. In a large review of 12,485 autopsy cases, only 154 secondary tumors (1.25%) and 7 primary cardiac tumors (0.056%) were identified [5]. More than one third of cardiac metastases originate from lung cancer, with approximately 20% arising from non solid primary malignancies such as lymphoma, leukemia and Kaposi sarcoma, 7% from breast carcinoma and 6% from carcinoma of the esophagus [1, 6]. Common routes of metastasis include direct invasion, hematogenous seeding, lymphatic spread, and intracavitary extension from the inferior vena cava [7]. Primary cardiac tumors are rare, and only about 15% of the primary cardiac tumors are malignant [8, 9]. Of the malignant tumors, the vast majority are sarcoma, followed by cardiac lymphoma and pericardial mesothelioma [10].

Cardiac tumors have a wide range of clinical manifestations. Its signs and symptoms are generally determined by its location and size, rather than its histology type [6]. Invasive tumors can cause valvular dysfunction, conduction abnormalities, and impaired myocardial contractility [10]. Tumors can embolize systematically to cause pulmonary embolism and end-organ failure. This case is an extreme example of cardiac tumor invasion presenting with a multitude of cardiac symptoms, including new-onset atrial flutter, pulmonary embolism, and obstructive right heart hemodynamics.

The initial diagnostic part of the case was uniquely challenging. Our initial endomyocardial biopsy via internal jugular vein was not diagnostic due to the profound tissue necrosis in the left atrium. An alternative route of biopsy needed to be explored. The standard approach to endomyocardial biopsy uses a transvenous approach that was first developed in 1962 when the Konno-Sakakibara bioptome was invented [11]. Since then, this technique has been refined and improved to become the standard practice for diagnosis of infiltrative cardiomyopathies and surveillance of heart transplant rejection. A handful of case reports have demonstrated the feasibility of both right atrial and left atrial masses biopsy using the EUS-FNA approach for diagnosis of cardiac sarcoma, cardiac lymphoma, and cardiac myxoma [12, 13]. While both procedures carry potential risks, in theory cardiac EUS-FNA has advantages over transvascular biopsies, such as real-time targeting of the lesion, avoiding damage to the valves or the coronary arteries during puncture, faster performance, and elimination of the need for fluoroscopy [13]. This case highlights the importance of considering various approaches to endomyocardial biopsy and confers both the feasibility and safety of utilizing endoscopic ultrasound guidance for left atrial biopsy.

Management of atrial masses is generally surgical if tumors are benign or resectable without evidence of metastasis [14]. Based on the histopathologic finding of this case, the patient’s cardiac mass is likely a metastatic disease from a primary pulmonary lesion, presumably too small to be detected by MRI or PET. However, further evaluation via sputum cytology or bronchoscopy was not performed at the time. Small cell carcinoma presenting as a cardiac mass has only been described in a handful of case studies [1–3, 15] with the majority documenting a primary pulmonary lesion. An extrapulmonary small cell carcinoma of cardiac origin would be incredibly rare, with only one potential case ever reported in the literature [2].

The backbone of managing metastatic small cell carcinoma is a combination of systemic chemotherapy with cisplatin or carboplatin and etoposide [16]. Recently, the addition of atezolizumab, a monoclonal antibody against PD-L1, has been approved by the U.S. Food and Drug Administration to be used in combination with carboplatin and etoposide as first-line therapy [17]. In extensive metastatic disease, radiation therapy can be used to treat symptomatic sites such as the brain, bone, or spinal metastasis. The role of chest radiation for metastatic small cell carcinoma presenting as cardiac mass is not clear but has been reported in one case report showing tumor regression without adverse events [3]. While surgical resection, chemotherapy or radiation therapy may be used for palliation in some cases, the overall prognosis for malignant cardiac tumors, both primary and secondary, is poor [15]. The prognosis of pulmonary small cell carcinoma is also poor with a 5-year survival rate of 6.3% [18].

With the extensive invasion of cardiac tissue by the tumor, treatment planning required us to weigh the benefits of chemotherapy for disease palliation against its potential adverse effects on the heart’s structural integrity. Although small cell carcinoma is an aggressive disease, it is initially quite sensitive to chemotherapy and radiotherapy [16]. The main cardiac risks of treatment included myocardial free rupture or development of a large atrial septal defect with lysis of the tumor, leading to rapid cardiac decompensation. As such, the patient was monitored in the intensive care unit while receiving his first round of chemotherapy.

We describe a rare occurrence of small cell carcinoma presenting as a massive cardiac tumor without apparent primary malignancy. Due to the tumor’s extensive invasion into the bilateral atria, interatrial septum and pericardium, alternative biopsy strategies and treatment considerations needed to be pursued. These options may inform the care of future patients presenting with an extensive malignant cardiac mass.

## Data Availability

Not applicable.

## Abbreviations

CT: Computed tomography
PET: Positron emission tomography
MRI: Magnetic resonance imaging
EUS-FNA: Endoscopic ultrasound guided fine needle aspiration
